# Screening Practices for Disordered Eating in Paediatric Type 1 Diabetes Clinics

**DOI:** 10.3390/nu13114187

**Published:** 2021-11-22

**Authors:** Emma Hanley Burden, Melissa Hart, Kirrilly Pursey, Peter P. Howley, Tenele A. Smith, Carmel E. Smart

**Affiliations:** 1 College of Health, Medicine and Wellbeing, The University of Newcastle, Callaghan, NSW 2308, Australia; Emma.Jones@uon.edu.au (E.H.B.); mel.hart@health.nsw.gov.au (M.H.); Kirrilly.Pursey@newcastle.edu.au (K.P.); tenele.smith@uon.edu.au (T.A.S.); 2 Hunter New England Mental Health Service, Waratah, NSW 2298, Australia; 3 School of Information and Physical Sciences/Data Science and Statistics, The University of Newcastle, Callaghan, NSW 2308, Australia; peter.howley@newcastle.edu.au; 4 Hunter Medical Research Institute, New Lambton Heights, NSW 2305, Australia; 5 John Hunter Children’s Hospital, Department of Paediatric Endocrinology and Diabetes, Newcastle, NSW 2303, Australia

**Keywords:** type 1 diabetes mellitus, feeding and eating disorders, early intervention, adolescents, Pediatrics, screening

## Abstract

Background: Type 1 Diabetes (T1D) is associated with increased risk of eating disorders. This study aimed to (1) assess adherence of Australasian paediatric T1D clinics to international guidelines on screening for disordered eating and (2) identify barriers and enablers to the use of screening tools for the identification of disordered eating. Methods: A 24-item survey covering five content domains: clinic characteristics, identification of disordered eating, screening tool use, training and competence, and pathways for referral, was sent to Australasian clinics caring for ≥150 children and adolescents with T1D. Results: Of 13 eligible clinics, 10 participated. Two reported rates of disordered eating of >20%, while eight reported rates < 5%. All clinics used the routine clinical interview as the primary method of screening for disordered eating. Only one used screening tools; these were not diabetes-specific or routinely used. Barriers to use of screening tools included shortage of time and lack of staff confidence around use (*n* = 7, 70%). Enablers included staff training in disordered eating. Conclusions: Screening tools for disordered eating are not utilised by most Australasian paediatric T1D clinics. Overall, low reported rates of disordered eating suggest that it may be undetected, potentially missing an opportunity for early intervention.

## 1. Introduction

Disordered eating behaviours include a wide range of unhealthy weight-management practices such as food restriction, compulsive eating and excessive eating which have not yet met the frequency or severity threshold for a diagnosable eating disorder [1]. Disordered eating is more common in children and young people with type 1 diabetes (T1D) compared to their peers without diabetes [2,3]. Without timely intervention this may progress to eating disorders. In a meta-analysis of children and young people aged 9 to 22 years, 7% of those with T1D had an eating disorder compared to 2.8% of people without T1D [3]. The focus on food as part of management and the increased burden associated with daily diabetes cares may contribute to the increased risk. Additionally, there is opportunity to engage in insulin restriction or omission to manage weight or achieve weight loss.

Having both T1D and an eating disorder is associated with poorer glycaemic control and higher risk of diabetes-related complications, including diabetic ketoacidosis [4], retinopathy, neuropathy and nephropathy [5], all of which contribute to increased morbidity and mortality [4,5,6,7]. To facilitate early detection, intervention and the prevention of eating disorders, routine screening for disordered eating in children and adolescents with T1D is recommended by international guidelines [7,8]. The American Diabetes Association recommends routine screening for disordered eating in children from 10–12 years using a diabetes-specific eating disorder screening tool, Diabetes Eating Problems Survey Revised (DEPS-R) [8]. The DEPS-R is a 16-item self-report screening tool for disordered eating validated for use in children, adolescents [9] and adults with T1D [10,11]. Other screening tools, including the mSCOFF have also been validated for use in the T1D population [12,13].

A number of international and Australian studies have suggested high rates of disordered eating behaviours in adolescents with T1D [14,15]. However, the processes surrounding the detection of disordered eating in paediatric clinics and potential barriers to guideline implementation have not been reported. The aim of this study was to explore current screening practices for disordered eating in Australasian tertiary paediatric T1D clinics and to identify barriers and enablers to the use of screening tools for the identification of disordered eating in the T1D population.

## 2. Methods

### 2.1. Study Design

A cross-sectional survey of Australian and New Zealand paediatric T1D clinics was conducted between June and September 2019. During this period, all eligible clinics were contacted via email and invited to complete an online survey. Information statements were provided. Completion of the survey was taken as consent. The study was approved by the Hunter New England Research Ethics Committee (2019/ETH00438). Eligibility criterion was clinics identified as managing ≥150 children and adolescents (0–18 years) with T1D annually; such clinics have been defined by international paediatric diabetes benchmarking as having ‘appropriate capacities for diagnosing, following up and managing patients’ [16]. For the purposes of this study, however, clinics were then asked to report on the number of children and adolescents aged 10–18 years as disordered eating is more relevant in this age group.

### 2.2. Survey Design, Dissemination and Retrieval

As there were no previously published or validated surveys of this kind, survey questions were initially developed for the study by a specialist team including a diabetes dietitian, an mental health dietitian, an eating behaviour researcher and a statistician representing a range of relevant expert knowledge and/or clinical experience. The survey was pilot-tested on eight clinical and research staff (who were not included as participants in the study). Staff were asked to comment on the scope of the survey content, i.e., if any questions were missing or were repeated as well as the clarity and flow of questions. In response to feedback, questions were removed and added and minor changes to the wording of questions were made to optimise clarity.

The final survey consisted of 24-items (a combination of open-ended (short-response), multiple response, single response and Likert scale questions) divided into five content domains: clinic characteristics and prevalence of eating disorders (7 items), pathways for treatment of eating disorders and disordered eating (3 items) identification of eating disorders and disordered eating (4 items), screening tool use, barriers and enablers (6 items) and staff screening tool awareness, training and competence (4 items). The survey took approximately 20 min to complete. 

The survey was generated using a web-based electronic data capture, management and survey tool, REDCap (Vanderbilt University, Nashville, TN, USA, 2009) and sent via a secure email link to a specialised diabetes dietitian at each eligible Australasian paediatric T1D clinic with instructions for completion by the interdisciplinary team. Each question required a response before proceeding to the next question. A copy of the survey is available on request.

### 2.3. Statistical Methods

Descriptive statistics and boxplots were used to reflect distributions of reported rates of adolescents diagnosed with an eating disorder or disordered eating in the past 12 months. JMP V14 (Cary, NC, USA: SAS Institute Inc.) was used for analyses. Theme and Pareto analyses were applied to open-response questions to identify methods and clinical indicators used to screen for disordered eating, as well as key barriers and enablers to screening in clinics.

## 3. Results

Thirteen clinics were eligible and approached to participate. Of these clinics, three failed to accept the invitation, representing a response rate of 77%. All dietitians reported that the survey was completed as part of a collaborative effort by the interdisciplinary team. Surveys were de-identified as per the requirements of the governing ethics committee, precluding comparisons of responding and non-responding clinics.

### 3.1. Clinic Characteristics

Participating clinics (*n* = 10) reported managing 106 to 1400 (median 282) children and adolescents aged 10–18 years with T1D. Although the eligibility criterion was management of ≥150 children and adolescents as per international benchmarking criteria [16], the lower numbers reflect the age range of 10–18 years, rather than from birth, given the ADA recommendations to commence routine screening for disordered eating from 10–12 years [8]. Across clinics, adolescents transitioned to adult care at a median age of 18 years, the youngest reported age for transition was 15 years. Team members included endocrinologists, diabetes educators, dietitians, psychologists, social workers and paediatricians. Less than half of all clinics (*n* = 4, 40%) had a psychologist working within the clinic. Of the six that did not have a psychologist, five had a social worker and one had neither a psychologist nor a social worker.

### 3.2. Prevalence of Eating Disorders and Disordered Eating

**Figure 1:**
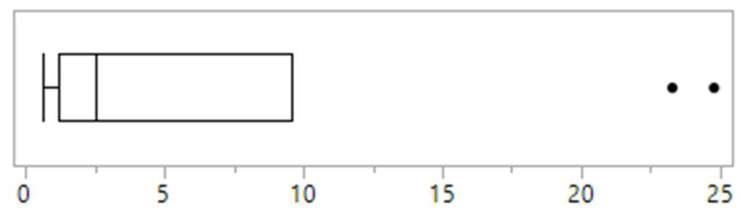
The proportion (%) of disordered eating reported by Australasian tertiary, paediatric type 1 diabetes clinics (*n* = 10). Vertical lines in boxplot (left to right) identify minimum, first quartile, median and third quartile values.

**Figure 2:**
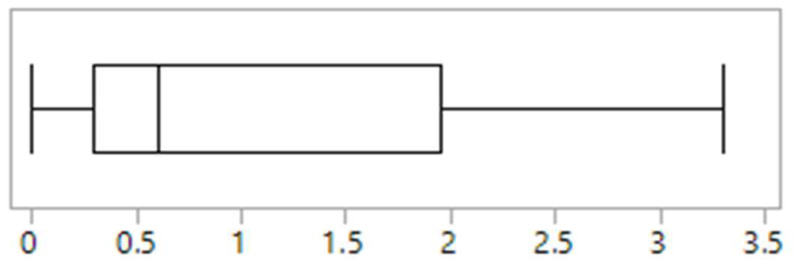
The proportion (%) of diagnosed eating disorders reported by Australasian tertiary, paediatric type 1 diabetes clinics (*n* = 10). Vertical lines in boxplot (left to right) identify minimum, first quartile, median, third quartile and maximum values.

Across clinics, the proportion of children and adolescents reportedly presenting with disordered eating within the previous 12 months ranged from 0.6% to 24.8%, with a median of 2.5% (Figure 1). The proportion of children and adolescents diagnosed and treated for an eating disorder ranged from 0% to 3.3% (Figure 2). The majority of clinics (*n* = 4, 40%) identified anorexia nervosa as the most commonly diagnosed eating disorder in their population, 30% (*n* = 3) identified other specified feeding and eating disorders, 10% (*n* = 1) identified avoidant restrictive food intake disorder and bulimia nervosa respectively, while 10% of clinics (*n* = 1) were unsure. No clinics identified binge eating disorder.

### 3.3. Screening Methods

One clinic (10%) reported using non-diabetes specific screening tools to identify disordered eating, including the SCOFF [17] and Eating Disorders Screening for Primary Care (ESP) [18]. These tools, however, were not routinely implemented and there was no procedure outlining their criteria for use. All clinics (*n* = 10, 100%) reported using a combination of clinical indicators, presented in Table 1 and behaviours detected during routine clinical interview to identify disordered eating. Admissions to hospital (*n* = 3, 30%), self-induced vomiting (*n* = 1, 10%) and parental concerns (*n* = 1, 10%) were also used to identify patients at risk of disordered eating. Half of all clinics (*n* = 5) reported that technology such as insulin pumps and continuous glucose monitoring had made a positive difference to the identification of disordered eating. The majority of clinics (*n* = 7, 70%) felt that the screening methods used in their practice were inadequate in identifying disordered eating. All clinics specifically noted that methods used to detect disordered eating relied on the experience of individual health professionals with the potential that there ‘would be many kids that go unnoticed’ or that ‘children would be missed due to not picking up signs, not asking the right questions during clinic review’. All clinics (*n* = 10, 100%) expressed that additional policies and procedures for screening and identification were needed.

### 3.4. Screening; Barriers and Enablers

The primary barriers to screening for disordered eating included time pressures (*n* = 9, 90%), absence of a screening tool or a tool clinicians felt was appropriate to use (*n* = 8, 80%), and lack of staff knowledge (*n* = 7, 70%) and confidence (*n* = 7, 70%) regarding the use of a screening tool. Most clinics (*n* = 9, 90%) declared that if barriers were addressed, they would routinely use a screening tool to identify disordered eating.

The most frequently cited enabler to the routine use of a screening tool for disordered eating identification was the availability of an appropriate validated screening tool (*n* = 6, 60%). One clinic stated that, ‘a simple, quick, discrete [tool] able to be used by a range of disciplines [is needed]’. Other enablers identified by health professionals included increased staffing (*n* = 2, 20%) and staff training in disordered eating (*n* = 2, 20%), more time in clinic appointments (*n* = 2, 20%), clarity around treatment referral pathways (*n* = 2, 20%) and having a psychologist embedded within the team (*n* = 1, 10%).

### 3.5. Staff Screening Tool Awareness, Training and Competence

Half of all clinics (*n* = 5, 50%) reported no previous training in eating disorder and disordered eating screening and assessment. The majority of clinics (*n* = 7, 70%) reported that staff awareness of screening tools for disordered eating was mixed, with some staff having high awareness but many low awareness. Two clinics reported all staff had low awareness, while one clinic reported all staff had high awareness. All clinics stated that competence of staff in detecting disordered eating varied, with some staff exhibiting low competency and others high competency. Most clinics (*n* = 7, 70%) reported that some, but not all staff perceived screening for eating disorders and disordered eating as a priority. Two clinics reported that all staff perceived screening as a priority, conversely, one reported all staff perceived screening as not a priority.

### 3.6. Characteristics of Clinics with Highest Prevalence of Disordered Eating

There was a clear delineation in the reported prevalence of disordered eating between clinics. Two clinics reported rates of 23.3% and 24.8%, while the remaining eight clinics reported rates of <5% (Figure 1). Of the two clinics reporting the highest rates of disordered eating, one was the only clinic that used screening tools and it was one of only four clinics that had a psychologist working in the team. This clinic also had the highest reported rate of eating disorder diagnosis (3.3%) (Figure 2). The other clinic with high reported rates uniquely had a social worker that was trained in eating disorders embedded in the referral process and who undertook routine screening for disordered eating as part of their role. This was the only clinic to report a high awareness of disordered eating amongst all staff, and one of only two clinics to report that eating disorder and disordered eating screening and identification was a high priority for all staff.

## 4. Discussion

International best-practice T1D clinical guidelines recommend the use of a screening tool for children, adolescents and adults with T1D to facilitate early identification and management of disordered eating and eating disorders [7,8]. Contrary to these guidelines, the current study demonstrates that routine screening for disordered eating with a screening tool is not currently undertaken in most Australasian paediatric T1D clinics and that diabetes clinicians feel they lack time, an appropriate tool and the necessary training in implementation of a tool. Instead, clinical markers detected during routine clinical interviews such as increase in HbA1c, insulin manipulation including insulin omission, change in growth trajectory including failure to grow, and behaviours such as restrictive eating, are the primary methods used for identification of disordered eating. In support of these, other studies have suggested the most common indicators of eating pathology in young people with diabetes are elevated HbA1c [6], disclosure of insulin misuse [6,19] and body image disturbance [20]. However, the concern remains that many of these clinical markers point to common diabetes management issues and that disordered eating in children and adolescents may be missed if an objective process for routine screening using a validated screening tool is not embedded in clinical processes.

There was significant variation in the reported prevalence of disordered eating and diagnosable eating disorders across clinics. This may be due to differences in identification practices. In two of the clinics surveyed, the estimated prevalence rates of disordered eating of approximately one in five children and adolescents was consistent with published literature in young adults [5,13]. However, eight clinics reported significantly lower rates (<5%). Overall, these findings are consistent with a previous chart review reporting that disordered eating was not adequately detected in paediatric patients during routine diabetes clinical visits, however, was commonly detected with a screening tool on transition to adult services. The low reported rates in Australasian clinics surveyed suggest that disordered eating in children and adolescents is not being identified, missing an opportunity for early intervention. This is an important finding given early identification and treatment for disordered eating may have the potential to alter the course, improve outcomes, and prevent diabetes complications [2,3,8,20].

Only one clinic reported the use of screening tools to identify disordered eating behaviour, the SCOFF [17] and ESP [18]. These tools are not diabetes-specific and may fail to account for unique aspects of T1D management, such as the need for an increased awareness of the amount of carbohydrate in foods and the potential for insulin omission and subsequently, may result in inaccurate representation of disordered eating in this population [20]. In contrast, the DEPS-R is a validated screening tool [9] that is recommended for use in children and adolescents with T1D [8] during routine appointments to screen for disordered eating behaviours [9]. It can be completed in less than 10 min [9]. However, no clinics reported the use of the DEPS-R and it is unclear whether this is due to a lack of awareness of the tool in paediatric T1D clinics in Australasia or the perceived difficulty of its clinical implementation. A recent systematic review conducted by our group has highlighted the current lack of validity and reliability of tools currently available to screen for and identify eating disorders and disordered eating in T1D [13]. This pilot study provides preliminary insights into the barriers and enablers to screening. Future directions include the development and implementation of consumer informed, time-effective frameworks incorporating validated, diabetes-specific screening tools and training in their use, alongside clinical indicators. However, to appropriately inform this work, further qualitative studies to elucidate provider perspectives on barriers and enablers to screening in the clinical setting are necessary.

The most common eating disorders diagnosed in T1D reported in the literature are bulimia nervosa and eating disorders not otherwise specified [7,21] In the present study, anorexia nervosa was the most commonly diagnosed eating disorder across clinics. While the population prevalence of anorexia nervosa is lower than other types of eating disorders, the apparent clinical manifestations and serious medical complications associated with anorexia nervosa may explain the higher rates of diagnosis. In addition, insulin omission is a disordered eating behaviour that can often go unrecognised, this may have contributed to the lower reported rates of other eating disorders such as bulimia nervosa.

The clinics reporting higher prevalence of disordered eating had higher staff awareness and perceived priority of identification of disordered eating, as well as mental health support in the team. International T1D guidelines currently recommend that mental health professionals with experience in paediatric T1D assess children and adolescents with T1D for psychosocial problems, including eating disorders [7,8]. The results of this study indicate this is needed in the Australasian setting along with increased staff training and knowledge regarding screening practices.

The present study has some limitations. The study was limited to clinics within Australia and New Zealand, and therefore, findings may not be generalisable beyond Australasia, although one US report similarly suggested that paediatric T1D clinicians may be missing cases of disordered eating [22]. The roles of, and number of team members participating in the survey at each clinic was not recorded and therefore, findings may not accurately reflect the experiences, perceptions and knowledge of all team members. Further, the brief nature of the open-ended questions meant that in-depth information regarding barriers and enablers to screening and which clinical indicators, in isolation or combination may prompt a clinician to conduct further assessment for the presence of disordered eating was not able to be elucidated. In addition, the survey was not validated; however, no validated survey exists, and the survey was developed with specialist input and then piloted.

## 5. Conclusions

This study identified that Australasian paediatric T1D clinics are infrequently employing validated screening tools to detect disordered eating in children and adolescents with T1D instead, relying on clinical indicators and behaviours detected during routine clinical interviews to identify at risk individuals. Training of diabetes health professionals to increase awareness of validated, diabetes-specific screening tools to identify disordered eating and to provide instruction on their use is warranted. This should be accompanied by health professional endorsed frameworks to support routine, systematic implementation of screening tools in clinical practice. There is also a need for clear referral pathways to enable timely access to supportive interventions.

## Figures and Tables

**Table 1:** Health professional identified clinical indicators to detect the potential for disordered eating.

Biochemical Markers (*n* = 10)	Change in Growth Trajectories (*n* = 9)	Insulin Manipulation (*n* = 5)	Dietary Behaviours (*n* = 5)
Increase in HbA1c Diabetic ketoacidosis Iron deficiency Abnormal lipids Large variation in interstitial glucose levels	Weight loss or excessive gain Delay in height growth	Insulin omission	Restrictive eating Omitting food groups Binge eating

## Data Availability

Survey available on request.
